# Construction and Application of a Human-Computer Collaborative Multimodal Practice Teaching Model for Preschool Education

**DOI:** 10.1155/2022/2973954

**Published:** 2022-06-24

**Authors:** Meimei Tuo, Baoxin Long

**Affiliations:** ^1^School of Education, Shannxi Fashion Engineering University, Xi'an, Shannxi 712000, China; ^2^School of Education, Shannxi Normal University, Xi'an, Shannxi 710062, China

## Abstract

This paper adopts the multimodal approach of human-computer collaboration to conduct an in-depth study and analysis of the practical teaching model of preschool education, and applies the designed model to the actual teaching process. The application of multimodal theory to preschool teaching is chosen to theoretically help expand the research scope of multimodal theory and enrich the research of preschool teaching, and practically help break through the previous single-modal teaching model, further enrich the theoretical guidance of preschool teaching, and improve the quality of preschool classroom teaching. Then, from the perspective of human-machine synergy, this paper analyzes the advantages of artificial intelligence technology and teachers in the English classroom, puts forward the new roles of teachers and learners in the human-computer cooperation teaching environment, and discusses the significance and value of applying the four main modules of human-computer cooperation teaching, human-computer gesture mapping and human-computer cooperation manipulator control in the preschool classroom. According to the physical structure of hand joints, the human hand joint angles are obtained through the inverse kinematic solution, and the human hand joint angles correspond to the dexterous manipulator one by one so that the dexterous manipulator can be controlled to imitate the human hand to complete flexible gesture movements and realize the vision-based collaborative human-machine control of the dexterous manipulator. Combined with Gagne's nine teaching events, a model of the English teaching process based on human-computer collaboration was constructed. Based on this model, the “EasyDotWise English Teaching System” was designed to combine the basic lesson types of preschool classroom teaching and the secondary objectives of the English curriculum standards, including “reading text–reading aloud evaluation,” “playing speech–sound recognition,” and “presenting text–selection.” We designed and implemented three types of teaching activities: “reading text–reading aloud assessment,” “playing phonetic sounds–sound identification,” and “presenting text–comprehension selection.”

## 1. Introduction

The hands have played a crucial role in the evolution of humans. Humans interact with the outside world, explore the world, and create it through their hands. In terms of human evolutionary history, the more things the hands can do and the more tasks they can undertake, the more powerful humans become in the evolutionary chain [1]. The production and use of tools are some of the differences between humans and animals. In ancient times, humans made and used tools with their hands, thus surviving on their wits under the rule of the weak and the strong. In the evolution of using in and out, the human palm and finger muscles were modified to adapt to increasingly complex and delicate manipulation tasks. Besides, sign language is the most natural body language that increases the expressiveness of the communicator and makes communication more efficient and smoother. Sign language is a special language for deaf people to communicate with the outside world and serves as a bridge for them to integrate into society. English classes based on modern educational technology bring a certain level of authenticity and contextualization to learners [2]. With the support of modern educational technology, learners get more information about sensory channels and representations through pictures, sounds, videos, and animations presented in the English classroom, which can establish rich connections to current things through the effective integration of the brain, and the information presented is closer to the information itself; therefore, the learning materials presented by using modern educational technology are more interesting, intuitive.

With the continuous combination of computer technology and educational methods, the application of modern multimedia e-learning facilities has become increasingly popular, which has led to the accelerated pace of Chinese teaching from unimodal to multimodal [3]. The teaching practices of many former international Chinese teachers prove that the traditional teaching model can no longer meet the learning needs of foreign Chinese learners in the new era, especially the needs of young Chinese learners, and the rapidly changing modern education technology provides a new opportunity for Chinese teaching reform.

The multimodal theory is precisely the use of modern educational tools, combining auditory, visual, tactile, and other senses, and teaching through a variety of means and symbolic resources such as language, images, sound, and movement, which is in line with the characteristics of Thai national education and the age characteristics of young learners [4]. At this stage, most English classroom teaching uses comprehensive English teaching software, and English teachers have difficulty in choosing appropriate and targeted technology according to learners' characteristics, resulting in using all functions. In addition, teachers mostly use interactive devices such as smart blackboards and all-in-one machines as blackboards in traditional classrooms, and rely excessively on modern educational technology, resulting in learners' inability to distinguish classroom priorities and put their focus [5]. This leads to the inability of learners to prioritize and focus on the application of technology, which leads to poor classroom results. This way of using technology for the sake of using technology can only keep the application of technology at the level of assistance, and it is difficult to realize the real cooperation between artificial intelligence and English teachers.

The learning paradigm based entirely on data will be difficult to get rid of the shackles of the law of large numbers and other statistical laws, and will not be able to achieve an efficient learning ability like that of human beings. In the future machine learning ecology, different learning tasks and learned models will not be isolated from each other. Knowledge migration between learning tasks and model reuse across tasks will become common, thus making learning models become learning components corresponding to software. This requires models that are not black boxes obtained from closed processes of learning, but open models with interpretability and transferability. The use of a series of intelligent technology products in the English classroom has enriched the content and mode of English classroom teaching, and to a certain extent has improved the teaching efficiency of the English classroom, but the use of technology also limits the development of teachers themselves [6]. How to balance the role of intelligent technology and teachers in the classroom and realize the “human-computer collaboration” teaching mode are important issues facing the technology-enhanced classroom teaching at present. To address the above problems, this study designs and develops an intelligent teaching assistant for English from the perspective of “human-computer collaboration” to solve the problems of speech input and language environment creation in English teaching. The assistant has the main functions of text-to-speech, automatic speech evaluation, listening materials production, etc. The intelligent teaching assistant assists English teachers to carry out classroom teaching, thus solving many problems in English teaching such as nonstandard speech input, promoting the modernization and intelligence of English teaching, and realizing the integration of artificial intelligence technology and English subject teaching [7].

## 2. Related Works

The scope of research on the application of artificial intelligence technology in English language teaching includes both horizontal and vertical aspects, with the horizontal scope referring to the types of education included in the research on artificial intelligence in English language education. The possible effects of using a humanoid robot as a teaching assistant for English vocabulary learning and memorization among Iranian foreign language learners with intellectual disabilities were investigated [8]. The study demonstrated the positive effects of using a humanoid robot to facilitate vocabulary learning and memorization in people with Down syndrome through a controlled experiment, and the results of this study may be a starting point for new research directions in foreign language teaching for people with down syndrome and reflect that technology has an important role in English language and teaching in special education [9]. This paper analyzes the changes of vocational education in economy, policy, ecology, and wisdom in s, and further points out the opportunities and difficulties faced by vocational education. The path of change and model reconstruction is needed in the teaching of English courses, environment, teacher development, evaluation, and educational management to meet the development goal of “leading artificial intelligence.” Individual interviews with faculty members of a degree program at a university in Madrid were conducted to determine faculty perceptions of the different approaches to the utility of the three intelligent access systems in learning activities, with the results indicating that bilingualism and selective conceptual interpretation were the features most valued by faculty members and that they found the incorporation of these systems in learning activities to be very useful.

Using this model, teachers can conduct targeted English teaching activities based on the optimization results of the system's algorithm and deepen the information about students' characteristics as a basis for optimizing English teaching strategies [10]. From the perspective of social semiotic analysis, Zdanevych et al. not only analyzed the role of multimodal symbols such as images, sounds, and colors in discourse but also studied the relationship between modality and media, proposing that media refers to the material resources used in the production of symbolic products and events [11]. Kelpšienė uses multimodal learning theory as a guide to explore the vocabulary learning mode in mobile language learning using WeChat as a platform compared with the traditional paper-based media, in which the former can more fully engage learners' visual and auditory senses and increase the vividness of vocabulary learning [12]. Taking students' English learning as the entry point, Zheng et al. analyzed the data mining training technique for English majors based on the Apriori algorithm, using the algorithm to realize deep mining of English learning data, to discover the study habits closely related to poor English learning performance, the influence of students' revision on students' performance, etc. [13]. Ruiz-Gómez et al. focus on the English subject and designs a test model based on the English subject's intelligent tutor system, which includes two modules: question bank maintenance and personalized grouping. The grouping module can realize personalized grouping services and generate test papers based on learners' learning proficiency [14]. Teachers have two roles to assume at this stage. One is to guide learners. What teachers need to do at this stage is to guide learners to use the student-side voice interactive feedback device correctly to avoid learners making mistakes in voice input.

The cost of hiring workers is much lower than that of experts, but the accuracy of individual workers is significantly lower than that of experts because workers often do not have expert capabilities. The core idea of crowdsourcing is to use a “crowd” of workers to replace a small number of experts to achieve the desired markup accuracy by aggregating the markup results of multiple workers while still saving costs [15]. Thus, how to effectively control the cost and quality at the same time becomes the core research problem of crowdsourced markup acquisition. Crowdsourcing markup acquisition is usually divided into two phases: worker labeling and markup aggregation. In the worker labeling phase, task publishers usually publish tasks through online crowdsourcing platforms, such as Amazon Mechanical Turk, and specify task assignment and reward mechanisms. In turn, workers participate in the tasks online and engage in labeling work according to the specified mechanism. In the marker aggregation stage, after obtaining the workers' markers, the task publisher needs to use the workers' marker results through marker aggregation algorithms to finally obtain accurate markers. And after the large-scale application of deep neural networks, the research of using a convolutional neural network for gesture recognition is increasing and gradually becomes a hot spot of research. The method using deep learning has the characteristics of flexible use and more robust recognition, etc. Integrating the above domestic and foreign research results, the convolutional neural network has a good prospect in gesture recognition and human-machine cooperative control with its advantages in the field of image recognition and applications.

## 3. Analysis of Human-Machine Collaborative Multimodal Teaching

Multimodal teaching is a new term first introduced by the New London Group, which advocates multimodal teaching using music, images, videos, and games to engage students' senses such as hearing and seeing to achieve interactive teaching and learning. Multimodal pedagogy is proposed, and it is believed that multimodality exists in every interactive activity in the classroom. Teachers use multiple channels and teaching methods such as sound, pictures, and animations to attract students' interest in learning, and then enhance students' participation in multimodal teaching. Specifically, multimodality in teaching refers to the ability to use modern media technology to engage students' visual, auditory, tactile, kinesthetic, and other sensory modalities in classroom communication and interaction to construct meaning. However, in the multimodal classroom vocabulary teaching, teachers should pay attention to the appropriate choice of various modalities. The real use of modalities is to help students understand the vocabulary content, to avoid classroom design that is too fancy and distracting students' attention.

(1) $$\begin{array}{c} y_{l}=f\left(\sum_{i\in M}y_{l}*x_{ij}^{l}-b\right). \end{array}$$

**Figure 1:**
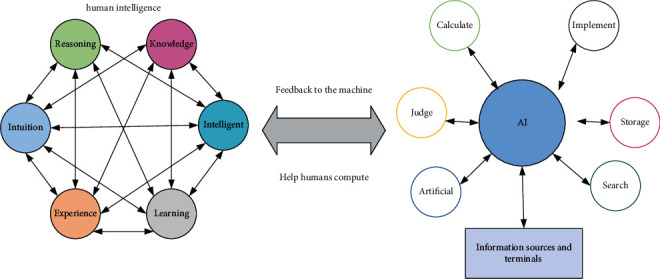
Human-machine collaborative intelligence system.

The multimodal theory, on the other hand, is inspired by systemic functional linguistics and agrees with the view that language is a social sign and meaning latent, and believes that language is not the only sign in the representational system but also nonlinguistic sign resources such as animation, dance, movement, facial expressions, and spatial distance are sources of meaning. The purely rational function hypothesis of systemic functional linguistics suggests that linguistic symbols can simultaneously perform three meta-functions in the process of expressing meaning, namely, conceptual function, interpersonal function, and discourse function [16]. The conceptual function refers to the use of language to represent the respective experiences in the objective and subjective worlds and to convey elements such as the time, place, and person of an event that are unknown to the listener. Interpersonal functions are those in which language reflects the speaker's status, identity, attitudes, motivations, and inferences about things. The discourse function refers to the function of interconnecting and organizing the linguistic components containing information into discourse, as shown in Figure 1.

Human-machine collaborative intelligence is an advanced application of research related to hybrid intelligence and human brain mechanism revealing, and it is also an inevitable trend in the development of hybrid intelligence research. Human-machine collaborative intelligence means that the human brain and the machine are completely integrated, solving the key technical problems of the underlying signal acquisition, signal analysis, information exchange, information fusion, and intelligent decision-making, so that the human brain and the machine truly become a complete system. In the research method of human-machine collaborative intelligence, the expression of human intelligence is different. Some studies are expressed in the form of data, and the goal of human-machine collaboration is achieved by using the data formed by human intelligence to train machine intelligence models. This collaborative approach usually adopts offline fusion, that is, human intelligence cannot guide and supervise machine intelligence in real time.

At the stage of integration of AI technology and English classroom teaching, the combination of AI and human teachers is already a normalized phenomenon in English classroom teaching, and human-machine collaborative teaching is an advanced stage of AI education application. At this stage, AI technology extends the intelligence of the human brain mainly in the following aspects, so that AI and human teachers can both play their respective strengths and work together to eventually cultivate learners into well-rounded individuals.

(2) $$\begin{array}{c} h_{t}=h_{\max}+\frac{t+1}{b\left(h_{\max}-h_{\min}\right)},t\in \left[0,b\right]. \end{array}$$

In the extension of perceptual intelligence, machines recognize images, speech, and text through perception technology, such as English text recognition, real-time English translation, and speech evaluation, which are commonly used in English classroom teaching, and the processing of such information is relatively inefficient and difficult for English teachers, but with the support of artificial intelligence technology, learners can better perceive and recognize various kinds of daily life. However, with the support of AI technology, learners can better perceive and recognize various information in daily life to extend the perceptive ability and scope of learners.

(3) $$\begin{array}{c} w_{a}^{k}=h_{k}\sqrt[{r}]{a}. \end{array}$$

This is because relying on a single modality in communication is often insufficient to accurately and fully express meaning, so another or even other modalities are used for reinforcement and supplementary explanations, so that the listener can better understand the meaning of the speaker's discourse. The expansion of cognitive intelligence, cognitive information is mainly technology through the process of calculation, reasoning, and analysis to recognize the complex cognition beyond the appearance of things; in actual teaching, teachers' cognition of learners is more through their own experience and other subjective feelings. Such feelings are easily affected by time, events, and other aspects to make cognition biased, but with the support of artificial intelligence technology, teachers can collect, calculate, reason, and analyze data on learners' learning process to determine learners' learning behavior from both objective data and subjective feelings, and make better cognition of learners. However, with the support of AI technology, teachers can collect, calculate, reason, and analyze the data of learners' learning process, determine learners' learning behavior from both objective data and subjective feelings, and make better cognition of learners.

(4) $$\begin{array}{c} L\left(x,y\right)=\frac{1}{N}\left(L_{\text{con}}f\left(x,y\right)\right). \end{array}$$

Educational AI intelligence should be able to reflect the organic integration of AI + education, teachers must make scientific and accurate decisions through comprehensive and accurate data, and technology should provide teachers with accurate decisions, and the two should synergize with each other to maximize the advantages of AI in education. The purpose of the human-machine collaboration is also to solve complex problems by combining the respective advantages of human intelligence and artificial intelligence. With regard to the subject of English as a language discipline, artificial intelligence brings greater possibilities and English teachers and learners play new roles during human-computer co-teaching.

(5) $$\begin{array}{c} g\left(x\right)=\frac{\left(g_{i}^{x},g_{i}^{y}\right)}{\left(d^{h}\right)}. \end{array}$$

The total confidence error is the sum of positive and negative sample errors, and the results of both positive and negative errors are classified by SoftMax, where positive samples are used for target prediction and negative samples are used for background prediction [17]. Since the SSD results produce a lot of prediction boxes, and most of the boxes are negative samples, the long tail effect is serious, and the positive and negative samples are seriously unbalanced, so the SSD does not use all the negative samples for training, first the negative samples are ranked. According to the confidence level from largest to smallest, and then select the ones with a large confidence level, and end the selection when the ratio of positive to negative samples is adjusted to 3 : 1. This NMS method can greatly improve the training efficiency of SSD, reduce the processing time, and at the same time can reduce the risk of overfitting, and the method has become a common processing means for the later stage of target detection methods. It is necessary to conduct a detailed analysis of the actual things of the intelligent teaching assistant, obtain the data parameters required for the design of the physical structure, select an excellent database management system, and fully understand its storage structure, access methods, and functional services provided.

**Figure 2:**
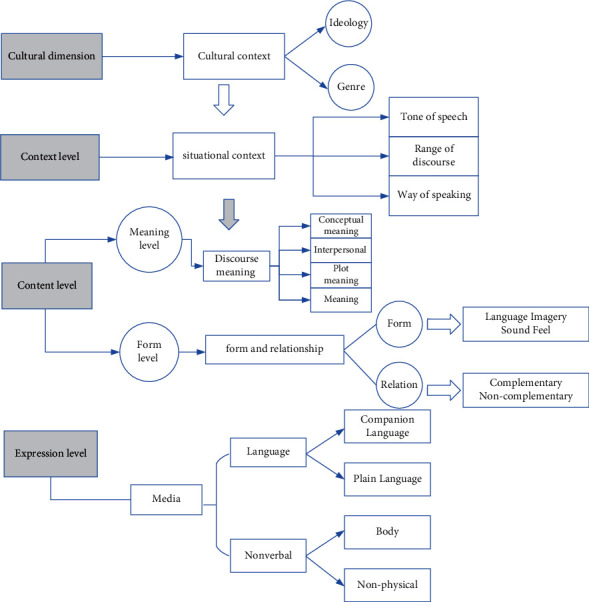
Integrated framework of multimodal theory.

In interpersonal communication, it is natural to use multiple modalities to express meaning, because communication often relies on a single modality to express meaning accurately and adequately, and thus uses another or even multiple modalities to reinforce and supplement the meaning, so that the listener can better understand the meaning of the speaker's words. Teachers should consider the different relationships between different modalities when teaching. The selection and combination of teaching modalities are constrained by the specific teaching process, so when combining multiple modalities, teachers should choose as many effective teaching methods as possible, especially modern educational technology, to achieve better teaching effects; according to the principle of optimizing the selection of modalities, the simpler the selection and combination of modalities, the better. Therefore, the combination of modalities in teaching is carried out in the contradiction of optimizing the effect and simplifying the implementation, as shown in Figure 2.

The framework is divided into four dimensions, the cultural dimension, the situational dimension, the content dimension, and the expression dimension. The cultural level is the key level that makes multimodal communication possible. The cultural dimension includes the ideology of people's thinking patterns, philosophies, and habits, which determine the traditions, forms, and technical support of communication. In the communicative process, communication is subject to situational context, which determines the meaning of discourse. Thus, the scope of discourse, the tone of discourse, and the mode of discourse govern the conceptual meaning, the interpersonal meaning, and the schematic meaning. At the formal level, different modal formal features are interrelated and work together to embody meaning. After the physical structure design is completed, the designed physical structure needs to be evaluated to obtain the use efficiency of the structure in the two dimensions of time and space. If it cannot meet the needs of the intelligent teaching assistant, the physical structure needs to be redesigned. Design and modify until the basic needs of an intelligent teaching assistant are met.

(6) $$\begin{array}{c} \sin\;\alpha =\left|MP*n\right|\frac{\sin\;x}{\cos\;x}. \end{array}$$

The levels of expression are the linguistic and nonlinguistic media. The whole framework not only demonstrates the expression, determination, and embodiment relationships of each level but also shows the equal relationship and interactions between language and other modalities. In multimodal teaching, teachers need to integrate and analyze the relationships among the four dimensions in the context of the multimodal theoretical framework and choose the most appropriate teaching modality according to the teaching reality.

Multimodal vocabulary instruction is a huge shift in the way of thinking about teaching strategies by changing from a single textual paraphrase to an instructional approach that makes full use of multimodal symbolic resources for meaning construction. However, in multimodal classroom vocabulary instruction, teachers should pay attention to the appropriate choice of various modalities and make the use of modalities to help students understand the vocabulary content to avoid the classroom design being too fancy to disturb students' attention.

**Figure 3:**
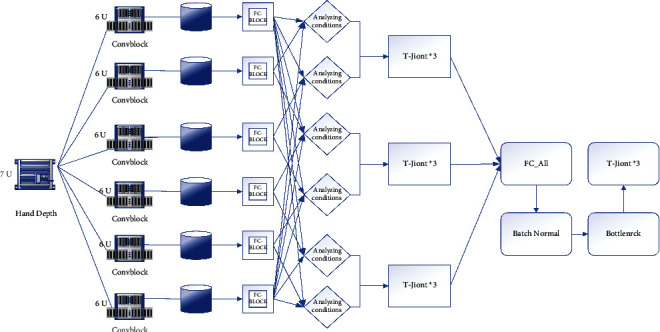
Overall diagram of branch convergence network.

After acquiring the human hand motion pose, it is necessary to map it with the motion parameters of the robot to control the robot motion by the human hand motion. The process involves two parts, one is the acquisition of the human hand pose parameters and the other is the assignment of the robot drive parameters. Therefore, the entire network does not depend too much on some local parameter node features, thereby enhancing the generalization effect of the SSD network model and reducing the risk of network overfitting. The joint parameters of the human hand are not only the result of hand joint tracking, because the physical structure of the human hand and the robot are not precisely corresponding, and it is impossible to assign the 3D position of the hand joint to the robot directly. Moreover, for the robot drive, it is generally assigned to the joint motor drive angle to make the robot joint reach the specified angle. Therefore, when obtaining the human hand motion parameters, it needs to be solved according to the requirements of the specific human-machine pose mapping method, as shown in Figure 3.

For pose mapping, a robot pose database needs to be established in advance, and the angle value of each joint is set for each pose. The human gesture is classified and estimated, and the currently detected human gesture category is matched with the gesture category in the robotic database so that the robotic hand can complete the specified gesture. The gesture mapping method is relatively simple to implement, only the classification of gestures is required, and different rules can be set for different kinds of manipulators, but the manipulator's action is limited by the number of inherent gestures, and only the set gestures can be completed.

(7) $$\begin{array}{c} \text{loss}=La+Lc+x*LaLc. \end{array}$$

It can be concluded that there is a clear relationship between the students' test scores and the teaching mode, and the multimodal vocabulary teaching mode is helpful to improve the scores. In terms of the hand itself, different fingers have different spaces of movement. The thumb has a unique para-palmar structure that plays an important role in gestural communication or manipulation of objects, and the thumb has the highest degree of freedom and the largest space of movement, so a separate branch is used to learn the characteristics of the thumb. Although the remaining four fingers are similar in structure and have the same degree of freedom, the index finger is the closest to the thumb and often cooperates with the thumb to form multiple gestures, thus the index finger is second in importance and a separate branch is also used to learn the features of the index finger. Considering that the muscular association between the middle finger and the ring finger constrains the movements of both to each other and they cannot move independently and flexibly in their natural state and that in many cases the middle finger, ring finger, and little finger only play a supporting role and have similar geometric structures and movement characteristics, these three fingers are grouped and their features are learned using a separate branching network.

(8) $$\begin{array}{c} L_{b}={\sum_{i=1}\left\|c_{i}+c_{i}\right\|}^{2}. \end{array}$$

Based on the above-observed analysis, the five-finger structure of the hand is reduced to three parts, corresponding to three branches of the network, and each branch network independently learns features specific to the corresponding hand region. The three branching networks are parallel and of equal importance, so that the feature extraction modules of each branch have the same structure. The cost of hiring a worker is much less than that of an expert, but since workers often do not have expert capabilities, the labeling accuracy of a single worker is significantly lower than that of experts.

Therefore, from the perspective of multimodal vocabulary teaching, the multimedia teaching resources can be fully utilized to imitate the real situation of interpersonal communication to the greatest extent possible and to engage students' multiple senses to make them feel like they are in the situation. To this end, teachers can use multimodal resources to design situational tasks and exercise students' ability to use vocabulary to effectively improve multimodal communication skills.

Multimodal scenarios can take a variety of forms, such as dubbing famous movies or news clips to expand vocabulary practice. Or teachers can use multimodal resources to set up a set scenario-based on vocabulary content and ask students to cooperate to simulate an interview or have a conversation; teachers can also design games to promote students' visual, auditory, and tactile senses to repeatedly practice relevant vocabulary in a relaxing atmosphere and deepen their impressions of vocabulary [18]. In this way, teachers integrate learning and entertainment into one, making English vocabulary teaching less tedious and practically fun.

## 4. Analysis of Preschool Education Teaching Model Construction Application

Multimodality exists in every interactive activity in the classroom. Teachers use sound, pictures, animation, and other channels, and various teaching methods to attract students' interest in learning in the classroom, thereby enhancing students' participation in multimodal teaching. By leveraging the wisdom of intelligent technology in concert with human teachers, it can help human teachers implement evaluation activities more objectively and rationally. At present, intelligent speech assessment technology has far surpassed human ability in terms of objectivity, quantification, and concurrent multiple evaluations, and intelligent speech assessment in English has been widely used in English-speaking examinations. Introducing intelligent speech assessment technology in English teaching and creating a collaborative human-computer evaluation method will be an important way to solve the problems of insufficient objectivity of evaluation and limitations in the scope of the evaluation.

**Figure 4:**
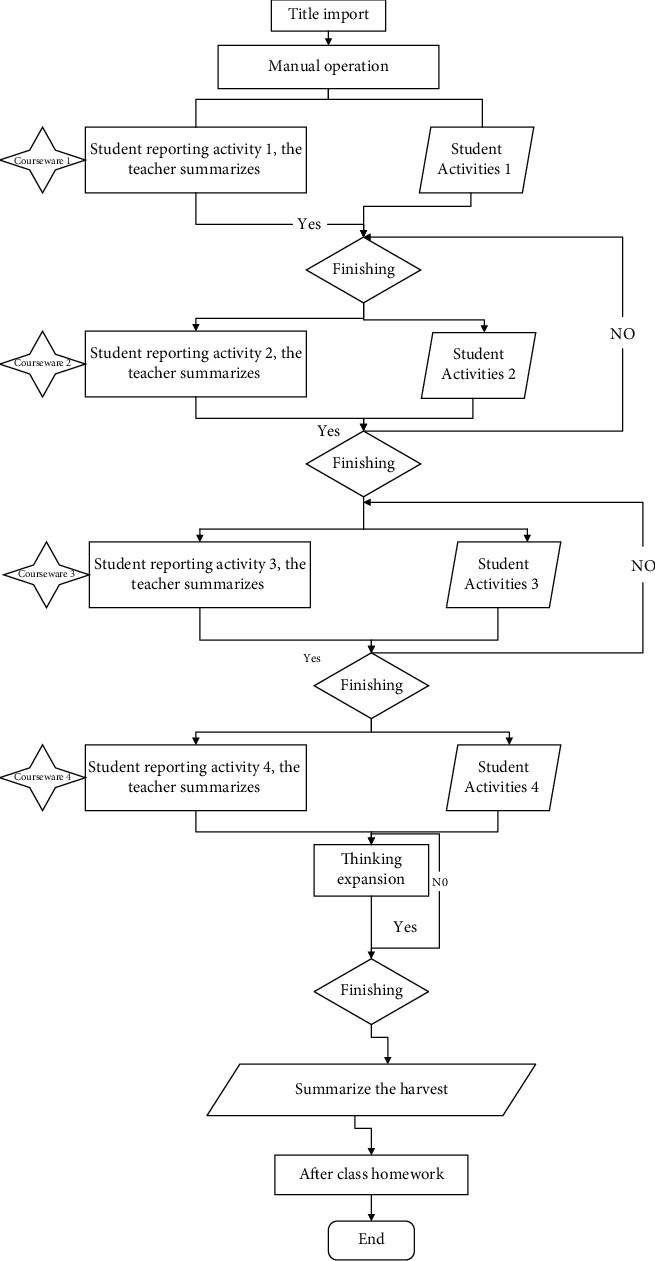
Flow of teaching activities.

The intelligent teaching assistant for English subjects needs to have English-speaking assessment function to assist teachers to implement assessment activities in an automated, objective, and quantifiable way. The quantitative assessment results are presented through visualization, and teachers will be freed from the tedious personal subjective assessment activities and focus on encouraging, communicating, and correcting the pronunciation of students. The voice assessment function of intelligent teaching assistants cooperates with teachers to carry out assessment activities, which can improve the objectivity of assessment, reduce the evaluation differentiation brought by teachers' single individual assessment, and realize the evaluation methods that are difficult to achieve in traditional classroom assessment. In the assessment function, the intelligent teaching assistant is used to assist teachers in assessing students, and the audio playback function is added to the assessment. Teachers only need to select students with low quantitative scores in the assessment, play their audio recordings, and make targeted pronunciation corrections. In this way, teachers can achieve the objective, quantitative, and timely feedback, which is difficult to be achieved by teachers, and free teachers from the complicated work to achieve both efficiency and quality improvement, as shown in Figure 4.

In addition to online resources such as video, audio, and PPT, teaching Chinese under multimodal theory also requires the use of physical teaching aids such as chalk, children's toys, and various handmade word cards. Therefore, teaching aids are an essential part of multimodal teaching [19]. As Thailand attaches great importance to the education of young children, most kindergartens are well equipped with teaching aids, which can better meet the requirements of multimodal theory for teaching equipment. Take the Sunflower Kindergarten in Bangkok, where the author works, as an example; it is a private kindergarten run by the private sector. The kindergarten has a separate dance classroom, music classroom, taekwondo classroom, etc. The teaching environment is good.

**Figure 5:**
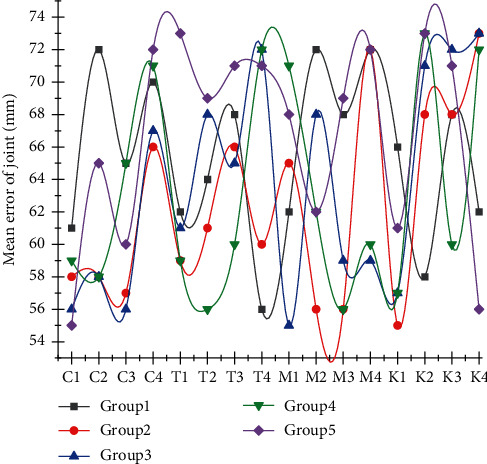
Self-contrast test of different branches.

The foreign language teachers' office is equipped with various stationery and teaching aids for teachers' use, and the office on the first floor is equipped with plastic sealers and printers, so teachers can print various teaching materials for free. Each classroom is equipped with modern teaching equipment for teachers to play teaching resources. Since the school adopts theme-based teaching, the school issues each classroom with teaching props for the corresponding theme every week, such as animal dolls, fruit models, etc. These teaching supplies are available for all teachers to use, as shown in Figure 5.

In carrying out the construction of a human-computer collaborative intelligent teaching and learning environment, the teacher clarifies which tasks he or she can accomplish and which ones require a lot of effort to accomplish or are impossible to accomplish personally. Therefore, the teacher has two roles to assume at this stage. One is to guide the learners, and what the teacher needs to do at this stage is to guide the learners to use the student-side voice interactive feedback device correctly to avoid learner errors in the voice input. In the specific teaching process in the classroom, teachers use Yidianhui to present the shape and pronunciation of key words such as factory worker, postal worker, businessperson, police officer, etc., and then explain the meaning of these words in detail. Let students understand vocabulary from the aspects of shape, sound, and meaning, and consolidate the memory of vocabulary.

In addition, teachers need to use words of encouragement to motivate learners to speak up and take the initiative to express their learning tasks in English. What teachers need to do at this stage is to select the appropriate functions of “eDotWise” according to the purpose of this teaching activity, i.e., to improve learners' pronunciation and intonation accuracy and their ability to express themselves in English. To improve the learners' pronunciation and intonation accuracy and their ability to express themselves in English, we select the intelligent evaluation function of “edgewise.” In this process, English teachers will abandon repetitive work and focus on guiding learners' thinking.

## 5. Performance Analysis of Human-Machine Collaborative Multimodal Teaching System

The purpose of the database logical structure design phase is to convert the conceptual model of the previous phase of conceptual structure design into a data model that can be supported by the database management system also called the logical model. The logical model can be used for the specific implementation of the database and is the model for the database management system. It is the data model supported by the specific database management system and is the data model that users can see in the database.

**Figure 6:**
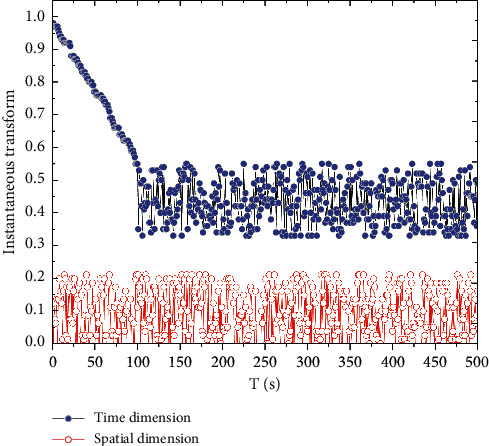
Immediate regret changes in synthetic data experiments.

The physical structure of the database of the intelligent teaching assistant in this study should meet the requirements of its short operation response time, high storage space utilization without redundancy, and high throughput of things [20]. Therefore, it is necessary to conduct a detailed analysis of the actual things of the intelligent teaching assistant, obtain the data parameters required for the physical structure design, select an excellent database management system, and fully understand its storage structure and access methods as well as the functional services provided. In addition, after the physical structure design is completed, the designed physical structure needs to be evaluated to obtain the efficiency of the structure in both time and space dimensions, and if it does not meet the requirements of the intelligent teaching assistant, the physical structure needs to be redesigned and modified until it meets the basic requirements of the intelligent teaching assistant, as shown in Figure 6.

**Figure 7:**
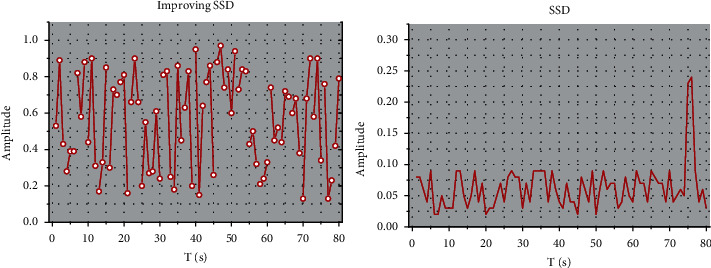
Improving SSD training and evaluation.

Dropout is used in the training process to solve the model overfitting problem by discarding the parameter weights of some intermediate nodes. Since the discarded node parameters are randomly selected after each iteration, the whole network does not depend too much on some local parameter node features, thus enhancing the generalization effect of the SSD network model and reducing the risk of network overfitting. The Batch Normalization technique is used to accelerate the convergence speed of the SSD network and improve the training effectiveness and efficiency of the SSD. The total loss value is 2.575 for the whole training process of 200000 steps, and the performance of the improved SSD model is evaluated using TensorFlow to see the performance on the test data, as shown in Figure 7; the evaluation result is 0.9686, which achieves a relatively good result. Teachers often use interactive devices such as smart blackboards and all-in-one computers as blackboards in traditional classrooms, and rely too much on modern educational technology, which makes learners unable to distinguish the priority of the classroom and focus on the application of technology, but the classroom effect is not good.

For OpenPose, this paper uses the official open-source hand keypoint detection model. The official open-source version uses the CMU Panoptic Dataset dataset from Carnegie Mellon University. The CMU Panoptic Dataset dataset uses 480 VGA cameras with a resolution of 640 × 480 pixels to acquire images at 25 frames per second, and in the same way, uses 31 cameras with a resolution of 1920 × 1080 pixels to acquire images at 30 frames per second, and uses 10 Kinect depth cameras that acquire images with depth information, 1920 × 1080 pixels, 512 × 424 depth, 30 frames per second, all clock synchronized to ensure time alignment. Five DLP projectors were also used to keep in sync with the HD devices. Compared to personal training, the official open-source version is not only more data-rich but also more accurate, making it far more effective in practice than personal training from scratch.

The 1 m distance test dataset is used to test the effect of adding the improved SSD on the original recognition accuracy and to verify the effect of the targeted adjustment of the prediction frame, while the 3 m distance test dataset is used to investigate whether the Open Pose model can increase the recognition distance and maintain the original accuracy after adding the improved SSD algorithm.

## 6. Teaching Model Application Results

**Figure 8:**
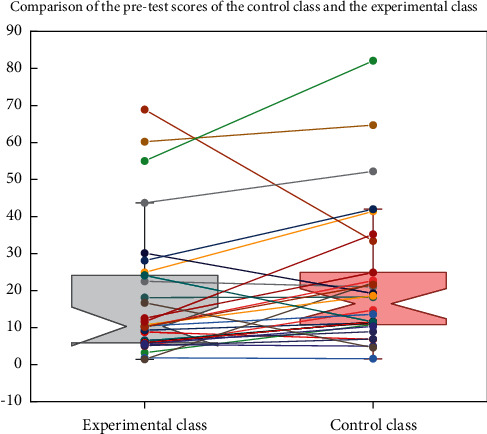
Comparison of the pre-test scores of the control class and the experimental class.

The data obtained from the pre-test, post-test, and questionnaire in this experiment were analyzed and processed using SPSS 17.0 data analysis software. According to Figure 8, the mean score of the pre-test in the control class was 78.922 scored and the mean score of the pre-test in the experimental class was 76.056 scored, and the difference between the mean scores of the two classes was not significant. To further verify whether there is a significant difference between the vocabulary scores of the students in these two classes, the researcher further conducted an independent samples *t*-test on this group of data.

Multimodal theory is to use modern educational methods, combine auditory, visual, tactile, and other senses, through language, image, sound, movement, and other means, and symbolic resources to teach, which is in line with the characteristics of Thailand's national education and suitable for early childhood learners. Age characteristics. According to the independent sample *t*-test of graph 8, the *p* value (Sig.) was 0.086, and the *p* value was greater than the significance level of 0.05, which also indicated that the achievement differences did not reach a statistically significant level, which means that there was no significant difference between the pre-test scores of the control and experimental classes. Therefore, it was reasonable to use these two classes as the subjects of the teaching experiment, and the experiment could be continued.

Taken together, there was almost no significant difference between the scores of the two classes before the experimental study, while after the teaching experiment, the mean test scores of the experimental class were higher than those of the control class, with a difference of 8.815 points between the two classes and a significant difference between the post-test scores of the two classes. Therefore, it can be concluded that there is a significant relationship between students' test scores and the teaching mode, and the multimodal vocabulary teaching mode helps to improve the scores.

To verify the effectiveness of the multimodal vocabulary teaching model, the researcher conducted a three-month experimental study on the experimental and control classes and administered a test to the experimental and control classes as well as a questionnaire to the experimental class to verify the effectiveness of multimodal vocabulary teaching and to find out students' attitudes toward multimodal vocabulary teaching. The ever-changing modern educational technology just provides a new opportunity for the reform of Chinese teaching. The learning materials presented by modern educational technology are more interesting, intuitive, and visualized, which can bring learners closer to real-life situations and improve their ability to use language skills in daily life.

**Figure 9:**
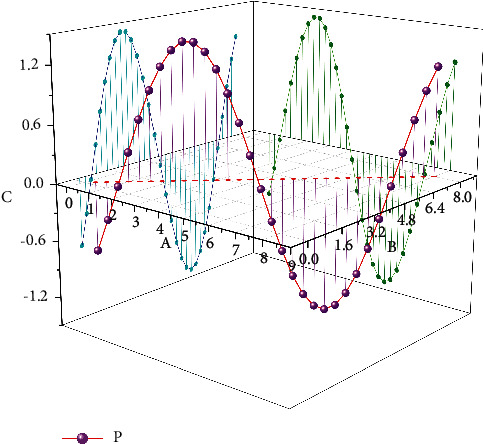
Frequency statistics of classroom coding behaviors of teaching activity cases.

Through the comparison of the means of the pre-test scores of the experimental and control classes and the independent samples *t*-test, as well as the data analysis of the questionnaire results, this study concluded that: The mean score of the pre-test of the control class was 78.922 scored, the mean score of the pre-test of the experimental class was 76.056 scored, and the mean score of the two classes was close, and the pre-test *p* value was 0.086, the *p* value was greater than the significance level of 0.05, which indicated that there was no significant difference between the pre-test scores of the control class and the experimental class; The mean score of the post-test was 84.870 0.086, and the *p* value was greater than the significance level, indicating that there was no significant difference between the two classes' pre-test scores; the mean 8.815 score of the post-test was 75.980 0.086, and the *p* ≤ 0.001, less than the significance level of 0.05. The multimodal vocabulary teaching model helps to improve junior high school students' English vocabulary performance, which shows that multimodal vocabulary teaching has received good results, as shown in Figure 9.

The teacher's speech act and the students' speech act accounted for 24.97% and 20.55%, respectively, indicating that the ratio of teachers' and students' interactive speech acts was relatively balanced. In the classroom, teachers present the shapes and pronunciations of key vocabulary such as factory worker, mail carrier, businessman, police officer, etc., and then explain the meanings of these words, so that students can understand the vocabulary in terms of shape, sound, and meaning, and consolidate their memory of the vocabulary.

The proportion of “silent behavior” in this lesson was 7.83%, of which 5.79% were “meaningful teaching pauses,” indicating that students had some time to think in this lesson, which helped learners to integrate new and old knowledge to a certain extent and complete the update of knowledge system. The percentage of “meaningless teaching pauses” is 2.04%, which is mainly due to the technical malfunction of the platform and the teacher's operation errors in the classroom-based environment, indicating that teachers should pay more attention to the details of teaching content operation and teaching platform use before class.

## 7. Conclusion

This paper uses multimodal theory as a guide to design instruction for preschool classrooms and summarizes the strengths and weaknesses of multimodal theory in teaching Chinese to young children in Thailand based on student feedback, classroom teacher feedback, supervising teacher feedback, and succession teacher feedback. Under the guidance of multimodal theory, the interest in Thai early childhood Chinese classrooms is greatly increased and students' learning effect is also improved. However, we cannot ignore the limitations of multimodal theory, such as students' attention shifting and classroom management problems, etc. Therefore, in response to the above problems, teachers need to choose modality flexibly and combine multiple modalities according to the specific conditions of their classes and schools, instead of relying too much on multimodal theory. Only by combining multimodal theory with teaching practice can the role of multimodality be better utilized and students can master the content more efficiently. Human-machine co-teaching can improve the teaching effectiveness of teachers and the learning effectiveness of learners. Through classroom observation and analysis of the three teaching activities and in-depth interviews with teachers and students, it can be found that teachers can accelerate teaching efficiency with the support of technology and make up for their pronunciation deficiencies to create a standard and authentic English language environment for learners. Learners can exercise their English language sense by perceiving the standard and authentic English language and strengthen their knowledge of English through the interaction between them and technology. The learners' learning initiative and information literacy will also be improved.

## Data Availability

The data used to support the findings of this study are available from the corresponding author upon request.
